# Electroacupuncture Pretreatment against Cerebral Ischemia/Reperfusion Injury through Mitophagy

**DOI:** 10.1155/2020/7486041

**Published:** 2020-09-09

**Authors:** Chenlu Mao, Cheng Hu, Yudi Zhou, Rong Zou, Sha Li, Yaomei Cui, Weiqian Tian

**Affiliations:** ^1^Department of Anesthesiology, Jiangsu Province Hospital of Chinese Medicine, Affiliated Hospital of Nanjing University of Chinese Medicine, Nanjing, Jiangsu, China; ^2^Affiliated Hospital of Nanjing University of Chinese Medicine, Nanjing, Jiangsu, China

## Abstract

Cerebral ischemia/reperfusion (I/R) injury can induce the mitophagy of neurons in the ischemic brain. Electroacupuncture (EA) pretreatment has a protective effect on cerebral ischemia/reperfusion injury. However, its internal mechanism still needs to be further studied. The present study's purpose is to investigate whether mitophagy is involved in neuroprotection elicited by EA pretreatment in a rat model of cerebral ischemia/reperfusion injury. The rats were pretreated with vehicle, EA at the Baihui (GV20) and Shuigou (GV26) acupoints 30 min daily, for 5 days consecutively prior to the focal cerebral ischemia injury induced by the middle cerebral artery occlusion (MCAO) model. Compared to the sham group, the neurological scores, infarction volume, number of autophagosomes, FUNDC1, p62, and the ratio of LC3-II/I were significantly increased but mitochondrial membrane potential and autophagy-related protein p-mTORC1 significantly decreased in the I/R group. However, EA pretreatment significantly reversed these trends. Overall, the results of this study demonstrated that EA pretreatment protected the cerebral ischemia/reperfusion injury which maybe correlated with mitophagy.

## 1. Introduction

The brain is the most sensitive organ of human to oxygen deficit. With the aging of the population, cerebrovascular disease has become the most common disabling and fatal disease worldwide, and the number of cases increased year by year, of which ischemic stroke accounts for more than 80% [1]. The injury of ischemic stroke is extremely harmful, which can cause severe neurological dysfunction and reduce the quality of life of patients. In the modern medicine concept, “prevention of disease” is one of important viewpoint. Therefore, it has a certain clinical value to reduce the incidence of stroke or the severity of cerebral ischemia/reperfusion (I/R) injury [2].

As an important alternative therapy, electroacupuncture (EA) is being accepted by more and more countries and regions. Previous studies have demonstrated that EA pretreatment can play a protective role in cerebral ischemic injury [3, 4]. After cerebral I/R injury, autophagy is significantly activated, which is manifested as autophagy of cytoplasm and mitochondria, aggravating neuron damage [5]. Autophagy is a process of maintaining homeostasis by lysosomal phagocytosis of necrotic organelles and misfolded proteins. Moderate autophagy plays a neuroprotective role, while excessive autophagy causes autophagic cell death [6]. Moreover, as the energy metabolism site of cells, mitochondria play a decisive role in the survival of cells.

Mitophagy, as one type of selective autophagy, is a selective process for maintaining the normal function of the organelle [7]. Mitophagy refers to the depolarization of mitochondria when cells are stimulated by damage, and the damaged mitochondria are specifically wrapped into autophagosome for degradation to maintain the stability of cell homeostasis [8, 9]. Damaged mitochondria can induce programmed cell death [10] and aggravate many diseases, including cerebral ischemia [11]. It is reported that cerebral I/R can induce the mitochondrial autophagy of neurons in the ischemic brain [12]. EA can improve the energy metabolism of mitochondria and reduce or reverse the damage of neurons after cerebral I/R in rats, which has a good neuroprotective effect [13].

As we all know, FUNDC1 is an integral mitochondrial outer-membrane protein and a receptor of hypoxia-induced mitophagy. The FUNDC1 contains three transmembrane domains and N-terminal LC3 interaction region, which plays an important role in mitochondrial autophagy [14]. It can interact with LC3 through its typical LC3-binding motif Y 【18】 xxL 【21】, and mutation of the LC3-interaction region impaired its interaction with LC3 and the subsequent induction of mitophagy [15].

Whether EA pretreatment affects the mitophagy in the process of regulating neurons autophagy induced by ischemia/reperfusion injury has not been illuminated yet. The exact mechanism of treatment is not well understood. Therefore, our study was designed to investigate the role of EA pretreatment in the protection of brain function against cerebral I/R injury using middle cerebral artery occlusion model rats, with a focus on mitophagy.

## 2. Materials and Methods

### 2.1. Animals

Male Sprague-Dawley rats, weighing 220–250 g, were provided by the Experimental Animal Center of the Nanjing University of Chinese Medicine and were housed under controlled conditions as follows: 12 h light/dark cycle, 22 ± 2°C, and 60–70% humidity, for a minimum of one week prior to EA pretreatment or surgery. All rats received a standard diet and free water. All procedures and assessments were approved by the Ethics Committee for Animal Experimentation of Nanjing University of Chinese Medicine (Nanjing, China) and performed in accordance with the National Institutes of Health Guidelines for Animal Research. There were 36 rats enrolled in this research, which were randomly divided into three groups: sham group (Sham); I/R group (I/R); EA pretreatment I/R group (EA + I/R). Each group contained 12 rats.

### 2.2. MCAO Model

Transient focal cerebral ischemia was carried out by MCAO. The environment temperature was kept at 25°C during the experiment. We followed the methods of Wu et al., 2013 [16]; all rats were anesthetized with an intraperitoneal injection of pentobarbital sodium (40 mg/kg body weight; Sigma-Aldrich, St. Louis, MO, USA). In 5–10 min after anesthesia, the rats were fixed in supine position. Then the right common carotid artery (CCA), the external carotid artery (ECA), and internal carotid artery (ICA) were exposed. A 2.0 monofilament nylon suture (Doccol Corporation, Redlands, CA, USA), with its tip rounded through heating in a flame, was inserted into the common carotid artery through an arteriectomy, just beneath the carotid bifurcation, and was advanced into the internal carotid artery ∼18–20 mm distal to the carotid bifurcation, until mild resistance indicated occlusion of the origin of the anterior cerebral artery and the MCA. Following 2 h of ischemia, reperfusion was accomplished by withdrawing the suture. In the sham group, the same surgery was performed, but without occlusion of the MCA.

Cerebral blood flow (CBF) of the MCA was measured using laser Doppler flowmetry. A flexible fiber-optic probe was affixed to the skull over the cortex supplied by the proximal part of the MCA (2 mm caudal to the bregma and 6 mm lateral to the middle). Rats exhibiting <80% reduction in CBF in the core of the MCA area were excluded from further investigation.

### 2.3. EA Pretreatment

EA pretreatment was performed at the Baihui (GV20) and Shuigou (GV26) acupoints. The Baihui and Shuigou acupoints are located at the intersection of the sagittal midline and the line between the two ears, 1 mm under the tip of the cleft lip, respectively. Animals were anesthetized and stimulated at an intensity of 1 mA, with a density-sparse wave for 30 min/day for five consecutive days prior to MCAO, using the Hwato Electronic Acupuncture Treatment Instrument (SDZ-V; Suzhou Medical Appliances Co., Ltd., Jiangsu, China).

### 2.4. Transient Focal Cerebral Ischemia

Rats were subjected to transient MCAO for 2 h following the final pretreatment as described above. Reperfusion was achieved by withdrawing the suture following 2 h ischemia. Cerebral blood flow (CBF) through the middle cerebral artery was measured using laser Doppler flowmetry (PeriFlux 5000; Perimed, Järfälla, Sweden). A flexible fiber-optic probe (PeriFlux 5000; Perimed) was affixed to the skull over the cortex supplied by the proximal part of the middle cerebral artery (2 mm caudal to bregma and 6 mm lateral to middle). Animals with <80% reduction in CBF in the core of the middle cerebral artery area were excluded from this study.

### 2.5. Neurobehavioral Evaluation

Neurobehavioral evaluation described by Longa et al. [17] was scored 24 h after reperfusion as follows: 0, no loss of nervous system function, normal activity; 1, failure to fully extend the front claw of the opposite side; 2, turning left when crawling; 3, dumping to hemiplegic side when walking; 4, unable to walk spontaneously with disturbance of consciousness. The higher the score, the more serious the animal behavior disorder.

### 2.6. Infarct Volume Assessment

Following decapitation, the brains were removed and frozen at −20°C for 15 min. The brains were then sliced using a plastic matrix (2 mm thickness; Sigma-Aldrich) and stained using 2% 2, 3, 5-triphenyltetrazolium chloride (TTC; Sigma-Aldrich) in 0.1 mol/l phosphate buffer (Sigma-Aldrich) for 30 min at 37°C to evaluate the infarct volume [18]. The infarcted tissue remained unstained (white), whereas the normal tissues were stained red. The infarct volume was calculated as follows: Infarct volume = (contralateral hemisphere volume-non-infarcted volume of the ipsilateral hemisphere)/contralateral hemisphere volume × 100%.

### 2.7. Ultrastructure Examination of Mitochondrial by Transmission Electron Microscopy

The rats were anesthetized with 40 mg/kg intraperitoneal pentobarbital sodium and then were perfused with precooled phosphate-buffered saline (PBS; pH 7.4), followed by PBS containing 4% paraformaldehyde (Sigma-Aldrich) and 0.25% glutaraldehyde (Sigma-Aldrich). The brain was cut in three slices, starting at the coronal plane of the frontal lobe, at a distance of three millimeters. The slices were 3, 4, and 3 mm thick from front to back, respectively. The intermediate sheet was then cut laterally from the centerline to a core made from a diagonal cut at the 2 o'clock position separated penumbra ischemic hemisphere 2 mm longitudinally. A 1 mm thick coronal slice from the cortex penumbra area was removed. The slice was placed in fresh prepared 2.5% glutaraldehyde overnight at 4°C. Following rinsing with 0.1 mol/l PBS three times, the slice was postfixed in 1% osmium tetroxide (Santa Cruz Biotechnology, Inc., Santa Cruz, CA, USA) for 1 h, dehydrated in graded ethanol (Sigma-Aldrich), and embedded in epoxy resin (Sigma-Aldrich). The polymerization was carried out at 80°C for 24 h. Blocks were cut from the slice using a Reichert/Leica Ultracut S Ultramicrotome (Leica Microsystems GmbH, Wetzlar, Germany) into ultrathin sections (60–70 nm), which were then poststained with uranyl acetate (Sigma-Aldrich) and lead citrate (Sigma-Aldrich) and examined using a Hitachi 7100 electron microscope (Nikon, Corporation, Toyko, Japan).

### 2.8. Western Blot Analysis

To analyze the expression of proteins, the rats were decapitated and brains were removed. The ischemic cortices and corresponding cortices of the sham rats were rapidly dissected. The mitochondria from the brain tissue samples were isolated using a mitochondrial fractionation kit (Genmed Scientific, Arlington, TX, USA), and the mitochondrial proteins were extracted. Protein concentrations were determined using a spectrophotometer (UV-2540; SHIMADZH Corp., Kyoto, Japan). A 20 *µ*g aliquot of protein from each sample was separated using 10% or 12% SDS-PAGE gels and subsequently transferred to a nitrocellulose membrane (Shanghai Haoran Bio-Technology Co., Ltd., Shanghai, China). The membranes were then incubated with specific polyclonal antibodies including LC3 (1 : 1,000; 3868S, CST), FUNDC1 (1 : 1,000, PAB23345, Abnova), p-mTORC1 (1 : 1,000; 14202S, CST), mTORC1 (1 : 1,000; 2983S, CST), ULK1 (1 : 1,000; 8054S, CST), and COX IV (1 : 1,000; ab33985, Abcam) or *β*-actin (1 : 1,000; Santa Cruz, USA) at 4°C overnight and then incubated with a horseradish peroxidase-conjugated goat anti-rabbit secondary antibody (1 : 2,000; ab6721, Abcam) at room temperature for 2 h. The membranes were washed again with TBST three times. The immune response was then purified by enhanced chemiluminescence (ECL) (Bio-Rad, USA). Immunoblot experiments were performed at least three times. Images were then used for final determination of protein expression using Image Lab™ software and normalized to the loading control.

### 2.9. Immunohistochemical (IHC) Analysis

Tissue sections were dewaxed, dehydrated, and rehydrated. Then, citrate buffer was used for antigen retrieval, and hydrogen peroxide (3.0%) was used to block endogenous peroxidase activity. After blocking with 10% goat plasma, primary antibodies, including an antibody against p62 (1 : 100, ab109012, Abcam), were added to the sections and incubated at 4°C overnight. Signal Stain Antibody Diluent (CST, US) was used to detect the primary antibodies. Counterstaining was performed using haematoxylin.

### 2.10. Detection of Mitochondrial Membrane Potential by JC-1

After 24 h of EA pretreatment and cerebral ischemia-reperfusion, the rats in each group were killed by anesthesia, soaked in a 70% alcohol for 2-3 sec, quickly removed, and placed in a super clean table, the scalp was cut off, the whole brain was carefully removed with surgical scissors and washed, the blood was removed with PBS, the hippocampal area in the brain was carefully separated and placed in a culture dish, and the tissues were filtered through 200 mesh and 400 mesh stainless steel mesh screens, and single cell suspension was prepared by PBS. After centrifugation and counting, about 6 × 10^5^ cells were resuspended in 0.5 ml culture medium, and 0.5 ml JC-1 staining solution was added. After mixing, the cells were placed in 37°C incubator for further incubation for 20 min. After incubation at 37°C, cells were centrifuged at 600 g 4°C for 4 min to precipitate. Discard the supernatant, wash it twice with JC-1 staining buffer (1×), add 1 ml JC-1 staining buffer (1×) to resuspend the cells, centrifuge at 600 g 4°C for 4 min, precipitate the cells, and discard the supernatant. Add 1 ml of JC-1 staining buffer (1×) to resuspend the cells, centrifuge at 600 g 4°C for 4 min, precipitate the cells, and discard the supernatant. After the cells were resuspended with JC-1 staining buffer (1×), then detect by flow cytometer (BD Calibur).

### 2.11. Statistical Analysis

The Graphpad prism 5.0 software was used to perform statistical analysis. Statistical significance was calculated using one-way analysis of variance (ANOVA) for multiple comparisons, while paired *t*-test was used to compare cerebral infarct volumes. The *P* value <0.05 was considered to indicate a statistically significant difference. All graphs show mean values ± SEM.

## 3. Results

### 3.1. EA Pretreatment Decreased Neurological Scores and Infarction Volume

**Figure 1:**
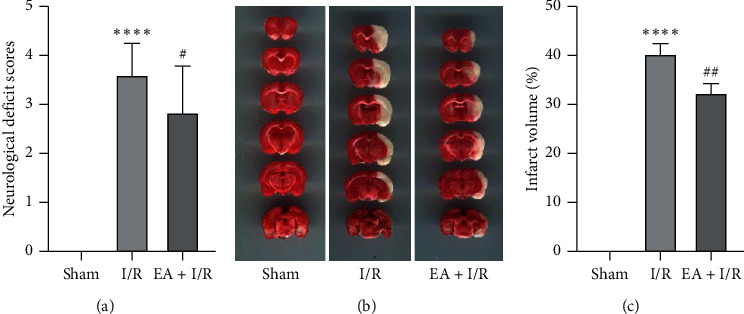
EA pretreatment decreased neurological scores and infarction volume. (a) Neurological function scores 24 h after ischemia/reperfusion injury in three groups; the higher the score, the more serious the animal behavior disorder and the worse the neurological function. ^*∗∗∗∗*^*P* < 0.0001 versus sham group; #*P* < 0.05 versus I/R group. (b) Representative images of rat brain with 2,3,5-triphenyltetrazolium chloride staining in three groups (red stain, normal brain tissues; white stain, infarct lesions). (c) Quantitative analysis of infarct volume in three groups (*n* = 6 per group). ^*∗∗∗∗*^*P* < 0.0001 versus sham group; ^##^*P* < 0.01 versus I/R group. I/R, ischemia/reperfusion; EA, electroacupuncture.

The neurological score of the I/R group was higher than that of the sham group. But the score of the EA + I/R group was lower than that of the I/R group (Figure 1(a)). The infarct area was evaluated by TTC staining in brain sections, with normal brain tissue in red and infarct brain tissue in white (Figure 1(b)). Compared with the sham group, the infarct volume of the brain increased significantly in the I/R and the EA + I/R groups. However, it reduced significantly in the EA + I/R group, compared with the I/R group (Figure 1(c)).

### 3.2. EA Pretreatment Improved the Microstructural Change

**Figure 2:**
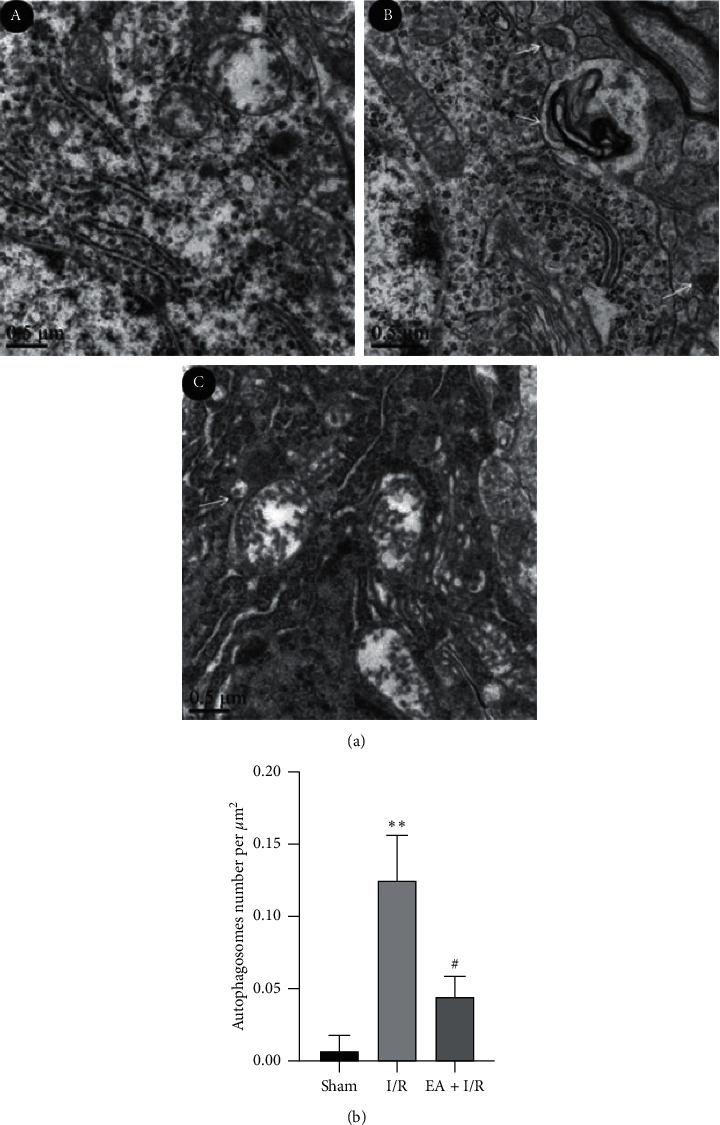
EA pretreatment improved the microstructural change. (a) The morphology of mitochondria observed by the electron microscope. Compared with the sham group, autophagosomes in the I/R group increased significantly, while the EA pretreatment group reversed this trend significantly (scale bar, 0.5 *µ*m). (b) The number of autophagosomes per *μ*m^2^ was quantitatively analyzed in different groups (*n* = 3 per group). ^*∗∗*^*P* < 0.01 versus sham; ^#^*P* < 0.05 versus I/R. I/R, ischemia/reperfusion; EA, electroacupuncture.

In the sham group, rough endoplasmic reticulum, mitochondria, and other organelles were normal in morphology, and no double-membrane autophagosomes were observed. In the I/R group, the number of mitochondria in the cytoplasm was reduced and the mitochondria were swelling. The endoplasmic reticulum became loose and formed many vacuoles. Moreover, the double-layer membrane structure of autophagic lysosomes was observed, involving the damaged mitochondrial structure. Neurons in the EA + I/R group displayed mild injury, with a number of normal organelles and nuclei observed; however, mitophagy remained present (Figures 2(a) and 2(b)).

### 3.3. The Autophagy-Related Proteins in Each Group

**Figure 3:**
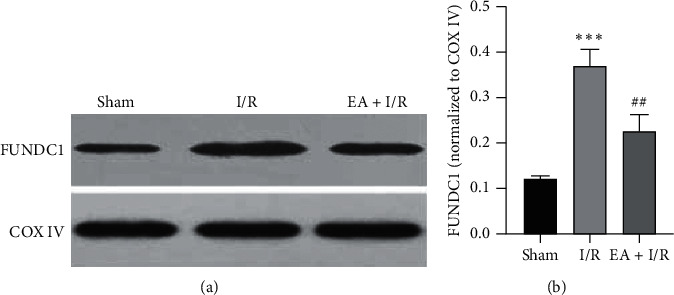
The expression level of FUNDC1 in each group. (a) The expression level of FUNDC1 in mitochondria was detected by western blotting and COX IV was used as a loading control; images acquisition was performed by Image LabTM software. (b) The quantitative analysis of FUNDC1 has also used Image LabTM software. The experiment was repeated three times and yielded similar results. ^*∗∗∗*^*P* < 0.001 versus sham; ^##^*P* < 0.01 versus I/R. I/R, ischemia/reperfusion; EA, electroacupuncture.

In order to study the mitophagy in different group, western blotting was used to detect the expression of FUNDC1 in mitochondria. Compared with the sham group, I/R led to a significant increase in FUNDC1. Due to the EA pretreatment, the expression of FUNDC1 decreased significantly (Figure 3(a)). Quantification of FUNDC1 by Image Lab™ software (Figure 3(b)).

**Figure 4:**
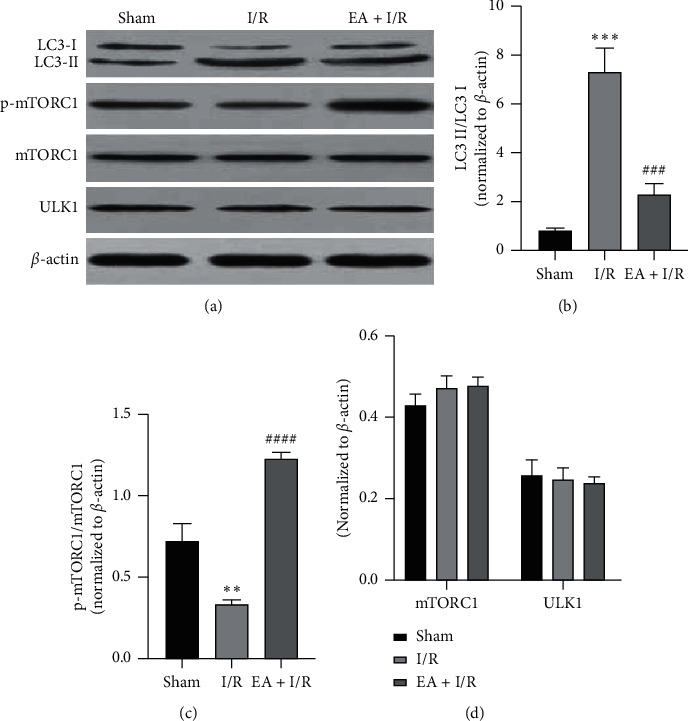
The autophagy-related proteins in each group. (a) Expression of LC3, p-mTORC1, mTORC1, and ULK1 in each group was detected by western blotting and *β*-actin was used as a loading control. (b–d) Quantitative analysis of the ratio of LC3-II/I and p-mTORC1/mTORC1 and the expression of mTORC1 and ULK1. The experiment was repeated three times and yielded similar results. ^*∗∗*^*P* < 0.01 and ^*∗∗∗*^*P* < 0.001 versus sham. ^###^*P* < 0.001 and ^####^*P* < 0.0001 versus I/R. I/R, ischemia/reperfusion; EA, electroacupuncture.

Compared with the sham group, the ratio of LC3-II/I increased but p-mTORC1/mTORC1 decreased in the I/R group. However, EA pretreatment significantly retarded this trend; the expression levels of mTORC1 had no significant changes in each group (Figure 4(a)). Quantification of LC3-II/I, p-mTORC1/mTORC1, and mTORC1 was by Image Lab™ software (Figures 4(b)–4(d)).

**Figure 5:**
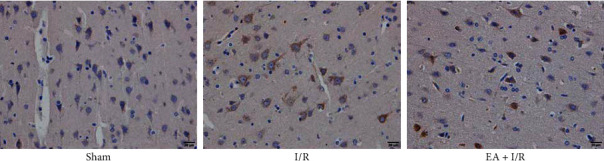
The expression of p62 in the different groups. The immunohistochemical staining was used to examine p62 expression in each group. EA pretreatment significantly increased the immunohistochemical staining expression of p62 compared with the I/R group.

Immunohistochemical staining showed the expression level of p62 in each group, as revealed in the immunohistochemistry observations; p62 in the EA + I/R group diminished compared with those in the I/R group (Figure 5).

### 3.4. Mitochondrial Membrane Potential Changes in Each Group

**Figure 6:**
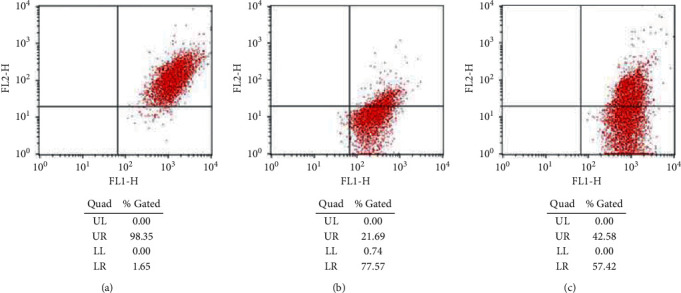
Mitochondrial membrane potential changes in each group. The JC-1 was used to examine mitochondrial membrane potential changes in each group. The fourth quadrant represents decreased mitochondrial membrane potential. (a) Sham. (b) I/R. (c) EA + IR.

The JC-1 was used to examine mitochondrial membrane potential changes in each group. In the I/R group, the mitochondrial membrane was potentially significantly reduced in comparison with sham group, while, in the EA + I/R group, membrane potential increased (Figure 6). EA pretreatment can improve autophagy of brain cells.

## 4. Discussion

The brain is the organ that consumes the most oxygen in resting state. As the main organ of energy metabolism, the number of mitochondria in the brain is much higher than that in other organs. However, due to the low energy storage in the brain, even a short period of ischemia and hypoxia may cause serious damage to the brain. Therefore, the brain is the most sensitive organ to hypoxia in human body. Current studies suggest that cerebral ischemia/reperfusion injury is related to the generation of free radicals, the toxicity of excitatory amino acid, the dysfunction of mitochondria, and the activation of apoptosis-related genes [19–22]. Mitochondrial dysfunction is an important link in cerebral I/R injury, and mitochondrial autophagy induced by mitochondrial dysfunction is also closely related to cerebral I/R injury.

Current studies have shown that EA pretreatment can reduce total cerebral I/R injury [23], and it is of great significance to clarify its mechanism. This experiment shows that EA reduced the cerebral infarct area and improved neurological function in rats caused by cerebral ischemia-reperfusion injury, which supports the view that EA pretreatment has significant benefits for cerebral I/R injury.

Autophagy is significantly activated after cerebral I/R injury, which is manifested as autophagy of organelles such as cell membrane, cytoplasm, and mitochondria, aggravating neuron damage [5]. Mitochondria are the main sites of reactive oxygen species (ROS) production, and they are more vulnerable to oxidative damage than other organelles. After mitochondria are damaged, a series of cascade signals will be triggered, thus initiating the occurrence of intracellular mitochondrial autophagy. In the process of regulating autophagy, whether EA pretreatment firstly affects autophagy of organelles such as mitochondria has not been reported yet. Our transmission electronic microscope results show that EA pretreatment can reduce the number of autolysosomes in rats with cerebral I/R injury model.

Recent studies [24] suggest that the mitochondrial protein FUNDC1 may mediate autophagy of mitochondria and play a key role in the selective clearance of damaged mitochondria. FUNDC1 is a highly conserved protein whose homologous proteins can be found in everything from low grade bacterial yeast to high grade primates, indicating that the function of this protein is very important. FUNDC1 is the outer membrane protein of mitochondria, which has three transmembrane domains, and the N-terminal is located in the cytoplasm, and the C-terminal is located in the gap between the inner and outer membrane of mitochondria. The N-terminal of FUNDC1 may interact with some other proteins related to autophagy, thus causing autophagy. High expression of FUNDC1 causes obvious mitochondrial autophagy and selective degradation of mitochondrial proteins. It was found in this study that I/R increased the expression of FUNDC1 and LC3-II/LC3-I ratio but decreased p-mTORC1/mTORC1, but EA pretreatment reversed this trend. At the same time, we used JC-1 staining to observe mitochondrial membrane potential and found that EA pretreatment can improve mitochondrial function and promote the survival of damaged neurons. Therefore, we believe that EA pretreatment can reduce cerebral ischemia/reperfusion injury by inhibiting mitophagy.

In conclusion, mitophagy plays a role in EA pretreatment of brain protection, and its signaling pathways need to be further studied.

## Data Availability

The data used to support the findings of this study were included in the article. All the authors declare that all the data in the manuscript were obtained by their experiments and the data are true and effective in the manuscript. The datasets used in the present manuscript are available from the corresponding author on reasonable request.

## Abbreviations

I/R: Ischemia/reperfusion
EA: Electroacupuncture
MCAO: Middle cerebral artery occlusion
LC3: Microtubule-associated protein 1 light chain 3
CCA: Common carotid artery
ECA: External carotid artery
ICA: Internal carotid artery
CBF: Cerebral blood flow
ROS: Reactive oxygen species
FUNDC1: FUN14 domain containing 1
TTC: 2, 3, 5-Triphenyltetrazolium chloride.

## References

[1] Truong D. T., Venna V. R., McCullough L. D., Fitch R. H. (2012). Deficits in auditory, cognitive, and motor processing following reversible middle cerebral artery occlusion in mice. *Experimental Neurology*.

[2] Zhang G. F., Yang P., Yin Z. (2018). Electroacupuncture preconditioning protects against focal cerebral ischemia/reperfusion injury via suppression of dynamin-related protein 1. *Neural Regeneration Research*.

[3] Wu Z. Q., Cui S. Y., Zhu L., Zou Z. Q. (2016). Study on the mechanism of mTOR-mediated autophagy during electroacupuncture pretreatment against cerebral ischemic injury. *Evidence-Based Complementary and Alternative Medicine*.

[4] Shu S., Li C. M., You Y. L. (2016). Electroacupuncture ameliorates cerebral ischemia-reperfusion injury by regulation of autophagy and apoptosis. *Evidence-Based Complementary and Alternative Medicine*.

[5] Yun Q., Jiang M., Wang J. (2015). Overexpression Bax interacting factor-1 protects cortical neurons against cerebral ischemia-reperfusion injury through regulation of ERK1/2 pathway. *Journal of the Neurological Sciences*.

[6] Ham O., Lee S. Y., Lee C. Y. (2015). Let-7b suppresses apoptosis and autophagy of human mesenchymal stem cells transplanted into ischemia/reperfusion injured heart 7by targeting caspase-3. *Stem Cell Research & Therapy*.

[7] Tang Y. C., Tian H. X., Yi T., Chen H. B. (2016). The critical roles of mitophagy in cerebral ischemia. *Protein & Cell*.

[8] Ashrafi G., Schwarz T. L. (2013). The pathways of mitophagy for quality control and clearance of mitochondria. *Cell Death and Differentiation*.

[9] Wei R., Cao J., Yao S. (2018). Matrine promotes liver cancer cell apoptosis by inhibiting mitophagy and PINK1/Parkin pathways. *Cell Stress & Chaperones*.

[10] Green D. R., Kroemer G. (2004). The pathophysiology of mitochondrial cell death. *Science*.

[11] Itoh K., Nakamura K., Iijima M., Sesaki H. (2012). Mitochondrial dynamics in neurodegeneration. *Trends in Cell Biology*.

[12] Zhou M., Xia Z. Y., Lei S. Q., Leng Y., Xue R. (2015). Role of mitophagy regulated by Parkin/DJ-1 in remote ischemic postconditioning-induced mitigation of focal cerebral ischemia-reperfusion. *European Review for Medical and Pharmacological Sciences*.

[13] Tian W. Q., Peng Y. G., Cui S. Y., Yao F. Z., Li B. G. (2015). Effects of electroacupuncture of different intensities on energy metabolism of mitochondria of brain cells in rats with cerebral ischemia-reperfusion injury. *Chinese Journal of Integrative Medicine*.

[14] Chen G., Han Z., Feng D. (2014). A regulatory signaling loop comprising the PGAM5 phosphatase and CK2 controls receptor-mediated mitophagy. *Molecular Cell*.

[15] Liu L., Feng D., Chen G. (2012). Mitochondrial outer-membrane protein FUNDC1 mediates hypoxia-induced mitophagy in mammalian cells. *Nature Cell Biology*.

[16] Wu X., He L., Cai Y. (2013). Induction of autophagy contributes to the myocardial protection of valsartan against ischemiareperfusion injury. *Molecular Medicine Reports*.

[17] Longa E. Z., Weinstein P. R., Carlson S., Cummins R. (1989). Reversible middle cerebral artery occlusion without craniectomy in rats. *Stroke*.

[18] Tsubokawa T., Jadhav V., Solaroglu I., Shiokawa Y., Konishi Y., Zhang J. (2007). Lecithinized superoxide dismutase improves outcomes and attenuates focal cerebral ischemic injury via antiapoptotic mechanisms in rats. *Stroke*.

[19] Kiirika L. M., Schmitz U., Colditz F. (2014). The alternative Medicago truncatula defense proteome of ROS-defective transgenic roots during early microbial infection. *Frontiers in Plant Science*.

[20] Borysov A., Krisanova N., Chunihin O., Ostapchenko L., Pozdnyakova N., Borisova T. (2014). A comparative study of neurotoxic potential of synthesized polysaccharide-coated and native ferritin-based magnetic nanoparticles. *Croatian Medical Journal*.

[21] Takarada T., Fukumori R., Yoneda Y. (2013). Mitochondrial uncoupling protein-2 in glutamate neurotoxicity. *Nihon Yakurigaku Zasshi. Folia Pharmacologica Japonica*.

[22] Schwartz C., Hampton M., Andrews M. T. (2013). Seasonal and regional differences in gene expression in the brain of a hibernating mammal. *PLoS One*.

[23] Zou R., Wu Z., Cui S. (2015). Electroacupuncture pretreatment attenuates bloodbrain barrier disruption following cerebral ischemia/reperfusion. *Molecular Medicine Reports*.

[24] Wei H., Liu L., Chen Q. (2015). Selective removal of mitochondria via mitophagy: distinct pathways for different mitochondrial stresses. *Biochimica et Biophysica Acta*.

